# Reliable and Scalable Identification and Prioritization of Putative Cellulolytic Anaerobes With Large Genome Data

**DOI:** 10.3389/fbinf.2022.813771

**Published:** 2022-03-17

**Authors:** Yubo Wang, Liguan Li, Yu Xia, Tong Zhang

**Affiliations:** ^1^ Environmental Microbiome Engineering and Biotechnology Laboratory, The University of Hong Kong, Pokfulam, China; ^2^ School of Environmental Science and Engineering, Southern University of Science and Technology, Shenzhen, China; ^3^ School of Environment and Energy, Peking University Shenzhen Graduate School, Shenzhen, China; ^4^ Shenzhen Bay Laboratory, Shenzhen, China

**Keywords:** bioinformatic pipeline, genome-centric, function interpretation, cellulose hydrolysis, anaerobes

## Abstract

In the era of high-throughput sequencing, genetic information that is inherently whispering hints of the microbes’ functional niches is becoming easily accessible; however, properly identifying and characterizing these genetic hints to infer the microbes’ functional niches remains a challenge. Regarding genome-centric interpretation on the specific functional niche of cellulose hydrolysis for anaerobes, often encountered in practice is a lack of confidence in predicting the anaerobes’ real cellulolytic competency based solely on abundances of the varying carbohydrate-active enzyme modules annotated or on their taxonomy affiliation. Recognition of the synergy machineries that include but not limited to the cellulosome gene clusters is equally important as the annotation of individual carbohydrate-active modules or genes. In the interpretation of complete genomes of 2,768 microbe strains whose phenotypes have been well documented, with the incorporation of an automatic recognition of synergy among the carbohydrate active elements annotated, an explicit genotype–phenotype correlation was evidenced to be feasible for cellulolytic anaerobes, and a bioinformatic pipeline was developed accordingly. This genome-centric pipeline would categorize putative cellulolytic anaerobes into six genotype groups based on differential cellulose-hydrolyzing capacity and varying synergy mechanisms. Suggested in this genotype–phenotype correlation analysis was a finer categorization of the cellulosome gene clusters: although cellulosome complexes, by their nature, could enable the assembly of a number of carbohydrate-active units, they do not certainly guarantee the formation of the cellulose–enzyme–microbe complex or the cellulose-hydrolyzing activity of the corresponding anaerobe strains, for example, the well-known *Clostridium acetobutylicum* strains. Also, recognized in this genotype-phenotype correlation analysis was the genetic foundation of a previously unrecognized machinery that may mediate the microbe–cellulose adhesion, to be specific, enzymes encoded by genes harboring both the surface layer homology and cellulose-binding CBM modules. Applicability of this pipeline on scalable annotation of large genome datasets was further tested with the annotation of 7,902 reference genomes downloaded from NCBI, from which 14 genomes of putative paradigm cellulose-hydrolyzing anaerobes were identified. We believe the pipeline developed in this study would be a good add as a bioinformatic tool for genome-centric interpretation of uncultivated anaerobes, specifically on their functional niche of cellulose hydrolysis.

## Introduction

Cellulose, being the dominant component of plant biomass produced in the process of photosynthesis, is the most abundant biopolymer on earth. Apart from quite few animal species such as termites and crayfish, capability of energy derivation from cellulose hydrolysis is known almost exclusively in microorganisms. Identification of putative cellulolytic microbes residing in varying natural habitats or engineered systems is a prerequisite for understanding the cellulose-hydrolyzing activities in the corresponding environmental niches. In this study, we focus specifically on genome-centric identification of putative cellulolytic anaerobes, which is important in contexts of nutrient synergism between gut microbes and ruminants (Svartstrom et al., 2017), energy recovery from cellulosic biomass through anaerobic digestion (Rubin, 2008), and industrial-scale solvent production (e.g., butanol) through fermentation of cellulosic biomass (Sayara and Sánchez, 2019). Traditional approaches, including microscopy observation and cultivation of cellulose-degrading microbes and purification and characterization of cellulolytic enzymes (Zuzana et al., 1994; Rastogi et al., 2009), have set a good foundation in understanding how the microbes and their enzymes may interact with substrates during cellulose hydrolysis. Although it is believed that most of the cellulolytic microbes, especially the anaerobes, may still be hiding in plain sight due to their isolation challenges, access to their genome information has opened a new window to shed light on them.

In the era of high-throughput sequencing, genetic information that is inherently whispering hints of the microbes’ functional niches is becoming easily accessible (Hug et al., 2016; Parks et al., 2017; Doud et al., 2020). Nevertheless, the challenge remains in properly identifying and characterizing these genetic hints to infer the microbes’ functional niches precisely. Regarding to the genome-centric prediction about the specific functional niche of cellulose hydrolysis, current approaches focus on tapping the diversity and the abundance of carbohydrate-active enzyme (CAZy) modules annotated in a genome: being subjected to the HMMsearch-based dbCAN platform (Yin et al., 2012) or the peptide-based CUPP (conserved unique peptide patterns) platform (Barrett et al., 2020), referring to the well-curated CAZy database (Cantarel et al., 2009; Yin et al., 2012), a decent amount of information on the abundances of each CAZy module annotated in a genome could be obtained. However, despite of these exciting progresses made for a more accurate/rapid annotation of individual CAZymes/CAZy modules, often encountered in practice is a lack of confidence in predicting the microbes’ real cellulolytic competency based solely on abundances of the varying CAZy modules/CAZymes annotated in their genomes. For example, a total number of 16 carbohydrate-active GH modules in the genome of *Spirochaeta smaragdinae DSM* 11293 could not point to a conclusion that this strain was able to hydrolyze cellulose (Mavromatis et al., 2010), and it remains a puzzle why *Clostridium acetobutylicum*, with the cellulosome gene cluster and 28 carbohydrate-active GH modules identified in its genome, does not have the cellulose-degrading capacity (Nolling et al., 2001; Bayer et al., 2004; Sreekumar et al., 2015).

What is missing in current genome-centric interpretation on the corresponding microbes’ cellulolytic competency is a recognition of the synergy among the varying carbohydrate-active units annotated, although synergism is one of the highly-appreciated features in efficient cellulose hydrolysis (Lynd et al., 2002; Gaston et al., 2016). Cellulolytic enzymes are known as modular proteins, and the most straightforward synergy would occur among the diverse CAZy modules co-occurring in one single gene or enzyme; for example, if one enzyme has both the cellulose-binding domain CBM6 and the exoglucanase domain GH48, the GH48 could be directed to its action site by its CBM6 partner (Bernardes et al., 2019). Based their co-occurrence patterns across genome and metagenome datasets, Konietzny et al. achieved targeted discovery of functional modules of plant biomass–degrading protein families (Sebastian et al., 2014).

A higher level of synergy being overlooked in current genome-centric annotation approaches is the synergy among diverse carbohydrate-active enzymes in the same microbe population, although cellulosome is the most highly recognized synergy machinery in anaerobes (Bayer et al., 2004; Doi and Kosugi, 2004). Comparing with cellulosome complexes in anaerobes, cellulose–enzyme–microbe (CEM) complex initiated by hypha penetration is the more commonly reported synergy machinery in aerobic cellulolytic *Fungi* (Lynd et al., 2002). Attachment of *Fungi* cells to its cellulosic substrate through hypha penetration would sandwich the extracellular carbohydrate-active enzymes in between the host *Fungi* cell and its cellulosic substrate, the way by which the CEM complex forms; physical closeness among the carbohydrate-active enzymes sandwiched in between the *Fungi* cell and its cellulose substrates makes the synergy among these enzymes possible (Akin et al., 1989; Akin et al., 1990; Tengerdy et al., 1991; Busto et al., 1996; Ho and Abdullah, 1999; Wilson, 2008).

Is the formation of cellulosome-independent CEM complexes possible in anaerobic bacteria (Dam et al., 2011)? To answer this question, checking whether anaerobic bacteria have alternative machineries that are analogous to *Fungi* hypha that could initiate the physical connection/adhesion of the anaerobe cell to its cellulosic substrate is necessary. We highlighted in this study the potential role of enzymes encoded by genes harboring both the surface layer homology (SLH) module and the cellulose-binding CBM (hereafter, termed as “cCBM”) module. Neither the SLH module nor the cCBM module is of hydrolytic activity, and the cell surface–anchoring role of the SLH module was reported in the formation CEM complexes through cellulosome complexes (Doi, 1999; Akihiko et al., 2002). From a theoretical point of view, enzymes encoded by these SLH-cCBM genes could adhere onto the microbes’ cell surface through its SLH domain, and the partnering cCBM domain could help drag the attached microbe cell to its cellulosic substrate; in other words, the SLH end of the enzyme may adhere onto the host microbe’s cell surface, and the cCBM end of the enzyme is cellulose-binding. Enzymes encoded by these SLH-cCBM genes may function as a “glue” to connect the host microbe cell with its cellulosic substrate, and such microbe–cellulose adhesion may sandwich the excreted enzymes in between the host microbe cell and its substrate, forming CEM complexes. It is reasonable to speculate that similar to the hypha-mediated CEM complex formation in *Fungi*, CEM complex mediated by these SLH-cCBM enzymes may provide the same physical closeness needed for synergy among enzymes aggregating in between the anaerobe cell and the cellulose substrate. Considering that most cellulolytic species are of optimal growth rates when they adhere to their cellulose substrate and the microbe-cellulose contact is important for the host microbes to get easy access to the enzymatic hydrolyzing products (Bayer et al., 1983; Raphael and Bayer, 1983), the role of enzymes encoded by these non-cellulosome SLH-cCBM genes in potentially mediating the CEM complex formation might have been overlooked, especially in anaerobes.

The objective of this study is to bridge the aforementioned research gaps by developing a bioinformatic pipeline that could 1) read the carbohydrate-active elements in the unit of genes that would facilitate recognition of the synergy among CAZy modules co-occurring in the same gene/enzyme; 2) identify and categorize putative gene apparatus that may initiate CEM complex formation in anaerobes, that is, the cellulosome gene clusters and the cellulosome-independent SLH-cCBM gene sets; and 3) to seek whether a more robust genotype-phenotype correlation might be achieved in the genome-centric identification of putative cellulolytic anaerobes by integrating into the current annotation approach an automatic recognition of the synergy machineries. The phenotype–genotype correlation analysis will be based on the interpretation of complete genomes of 2,768 microbe strains whose phenotypes have been well documented; and the application of this pipeline in scalable annotation of large genome dataset would be further tested with 7,904 reference genomes downloaded from NCBI.

## Materials and Methods

### Collection of Reference Complete Genomes and Reference Genomes

A total of 5,243 GenBank Format (GBK) files corresponding to 2,786 prokaryote strains with complete genomes were downloaded from the NCBI genomes_FTP_site (ftp://ftp.ncbi.nlm.nih.gov/genomes/archive/old_genbank/Bacteria/). Chromosome and plasmid of the same microbe strain correspond to separate GBK files, and metadata of these 5,243 GBK files of the 2,786 prokaryote strains could be found in Supplementary Appendix S2. Fasta amino acid (Weimer et al.) sequences of the coding regions (often abbreviated as CDS) in these 2,786 complete genomes were extracted from 5,243 GBK files with a custom python (version 3.7.5) script (the python script “*GBK_Parser_faa.py*” has been deposited in Github). Another 7,904 reference genomes were also downloaded from NCBI (ftp://ftp.ncbi.nlm.nih.gov/genomes/refseq/bacteria/) (updated on February 2020), and there are metagenome–assembled genomes (MAGs) among these 7,904 reference genomes. These 7,904 reference genomes were used to evaluate the time-efficiency of the scalable annotation of the large genome dataset, which is discussed in detail in the results section, and the metadata of these 7,904 reference genomes could be found in Supplementary Appendix S4.

### Genome-Centric Annotation of Carbohydrate-Active Enzyme Modules and Visualization of the Clustering Patterns of Carbohydrate-Active Enzyme Modules Along Genes

Fasta amino acid (Weimer et al.) sequences of the 2,786 complete genomes and the 7,904 reference genomes were subjected to dbCAN HMMsearch for the CAZy module annotation, applying the HMMsearch criteria (e.g. cutoff value) as recommended by the dbCAN annotation platform (Yin et al., 2012). The shell script (“dbCAN.sh”) written for the batch annotation of a large number of genomes has been deposited in Github. CAZy modules were identified in 2,642 of these 2,786 complete genomes, and by applying the custom R script “*complete_genome_annotation.R*” (R version 4.1.0, updated on 2021-05-18), abundance of the diverse CAZy modules annotated in each of the 2,642 complete genome was summarized; in cases where the chromosome and the plasmid in the same microbe strain correspond to separate FAA files, results of the annotation of those separate FAA files would be aggregated. Applying the custom R script “*MIMAG_annotation.R*”, abundance of the diverse CAZy modules annotated in each of the 7,904 reference genome was summarized.

Genes are the basic units encoding enzymes, and the most straightforward synergy is among the diverse CAZy modules co-occurring in one single gene or enzyme. Employing the genoplotR package (version 0.8.11) (Guy et al., 2010), referring to the position of each CAZy module annotated in genes, visualization of the clustering pattern of the CAZy modules along genes in each genome can be achieved with the custom script “*genoplot_CAZy_in_genomes.R*”. The file “NC_009012.pdf” deposited in Github acts as a demonstration of such visualization in the chromosome of strain *Ruminiclostridium thermocellum* ATCC 27405. All the R scripts written in this study have been deposited in Github.

### Calculation of the Frequencies at Which two Carbohydrate-Active Enzyme Modules Co-occur With Each Other in the Same Gene

To avoid potential errors that may originate from truncated genes in draft genomes, frequencies of CAZy modules co-occurring with one another in same genes were calculated from genes annotated in 2,642 complete genomes. Principles for such calculation could be illustrated with the example of frequencies at which CAZy modules co-occur with GH9 in the same gene: first, all carbohydrate active genes harboring the GH9 module were retrieved from the 2,642 complete genomes, and the total number of GH9 annotated in these genes was counted as “N”; second, among these GH9-harboring genes, the total number of each of the co-occurring CAZy module was calculated to be n_1_, n_2_, n_3_ …n_i_…., respectively; third, divide n_i_ by N, and ( $\frac{\text{ni}}{\text{N}}$ ) *100% would be taken as the frequency at which the CAZy module “i” being identified in the same gene as GH9. The custom script “CAZy co-occurrence frequency.R” written for such calculation of the co-occurring frequencies among the CAZy modules has been deposited in Github.

### Categorization of Carbohydrate-Active Genes According to Harbored Carbohydrate-Active Enzyme Modules

**FIGURE 1:**
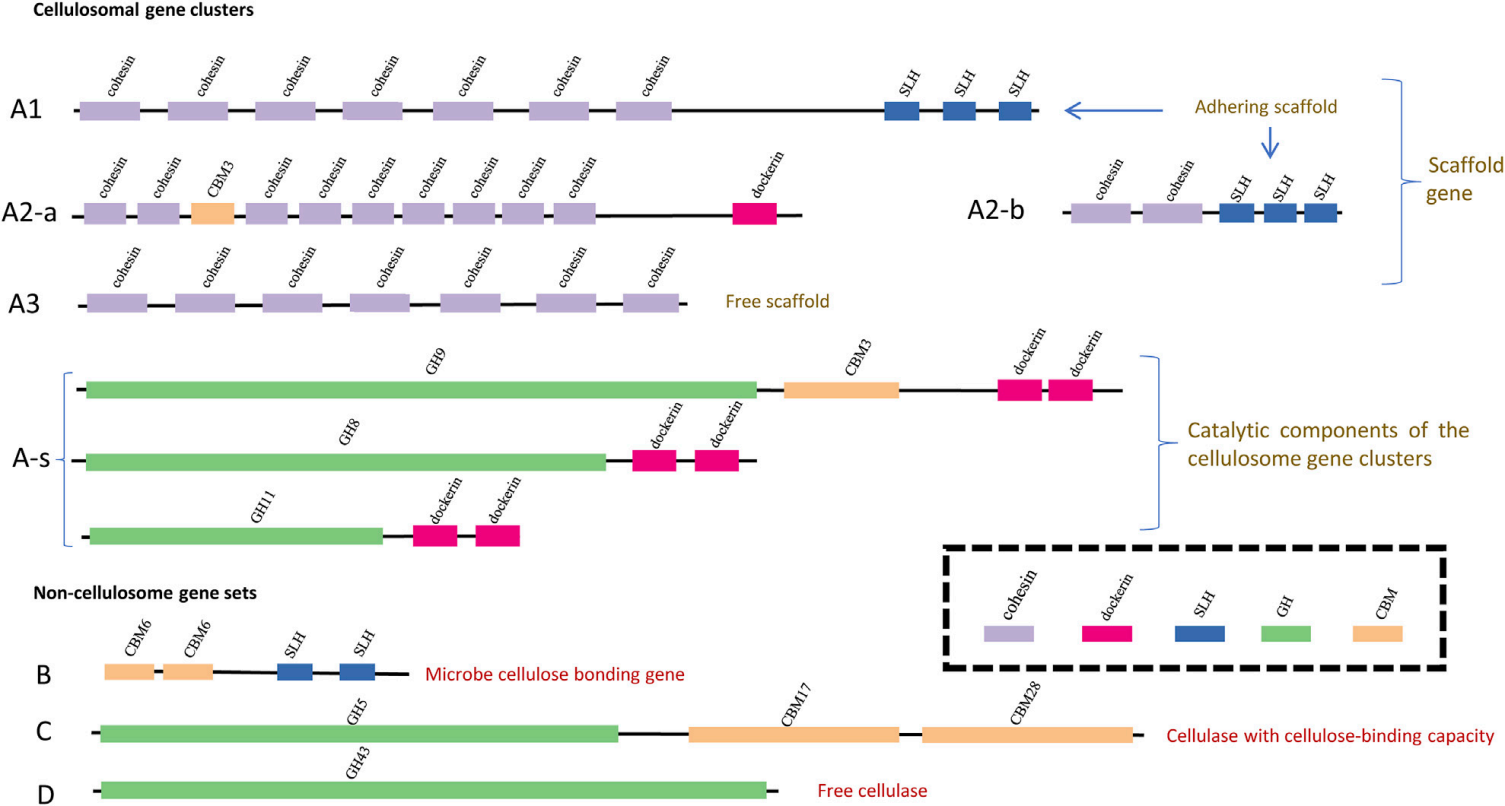
Schematic visualization of the clustering patterns of CAZy modules along different types of cellulose-active genes. The GH modules, the CBM modules, the SLH module, the dockerin module and the cohesion module are represented with bars in green, yellow, blue, pink and purple, respectively; width and position of each bar indicates the length (bp) of each module and its position along genes. Definition of the gene types are the same as that summarized in Table 1: genes of type **(A1,A2-a,A2-b)** are adhering scaffold genes; genes of type **(A3)** represent non-adhering scaffold backbone genes (missing the SLH module); genes of type **(A-s)** are the catalytic components of the cellulosome gene clusters; genes of type **(B)** encode the microbe-cellulose adhesion protein; genes of type **(C)** encode cellulase with cellulose-binding capacity and genes of type **(D)** encode free cellulase (missing both the CBM module and the SLH module).

To make synergy among CAZy modules in the same genes identifiable and countable, criteria were proposed in this study to recognize and categorize cellulose-active genes according to the co-occurring patterns of the CAZy modules they harbor, and these criteria are summarized in Table 1. Part of the visualization of CAZy modules co-occurring in same genes is demonstrated in Figure 1, and cellulose-active genes are classified into two broad categories: cellulosome gene clusters and non-cellulosome gene sets.

**TABLE 1:** Criteria proposed in this study to identify and categorize carbohydrate-active genes. A visualized version of this categorization could be found in Figure 2. “cCBM” in this table refers to cellulose-binding CBM modules (a list of these cCBM modules could be found in Supplementary Table S3), and “cGH” in this table refers to those GH modules with reported exo/endoglucanase activities or lytic polysaccharide monooxygenase (LPMO) activities (a list of these cGH and LPMO modules can be found in Supplementary Table S2).

Gene categorization			Clustering of the CAZy modules along genes					Description of the gene products
			SLH	Dokerin	Cohesin	cGH	cCBM	
Cellulosomal gene clusters	Scaffold backbone	A1	>=1	0	>=3	>=0	>=0	Scaffold with the SLH module
								(…+Cohesin+Cohesin+…+Cohesin+SLH+…)
		A2-a	>=0	>=1	>=3	>=0	>=0	Scaffold with a dockerin tail that could assemble the SLH modules from A2-b into its backbone
								(…+Cohesin+Cohesin+…+Cohesin+Dockerin+…)
		A2-b	>=1	>=0	>=1	>=0	>=0	SLH module to be assembled into the scaffold backbone of A2-a
								(…+Cohesin+SLH+…)
		A3	0	0	>=3	>=0	>=0	SLH-free scaffold without either a SLH module or a dockerin tail
								(…+Cohesin+Cohesin+…+Cohesin+…)
	Catalytic components	A-s	>=0	>=1	>=0	>=1	>=0	Catalytic components of the cellulosome gene clusters (…+Dockerin+GH/cCBM+…)
Non-cellulosome gene sets		B	>=1	>=0	>=0	>=0	>=1	Microbe-cellulose bonding gene
								(…+SLH+ cCBM+…)
		C	>=0	>=0	>=0	>=1	>=1	Cellulase with cellulose-binding capacity (…+GH+cCBM+…)
		D	>=0	>=0	>=0	>=1	0	free cellulase (solitude GH)

The cellulosome gene clusters consist of two parts: 1) scaffold backbone genes with at least three cohesion modules (“A”: cohesion + cohesion + … + cohesion +…) and 2) genes encoding the catalytic components that could be assembled into scaffold backbones (“A-s”: dockerin + GH/CBM). Among the cellulosome gene clusters identified in nine out of the 2,768 complete genomes, it was noted that not all cellulosome gene clusters harbor the cell surface–anchoring SLH module; the phenotype relevance of this subtle variation is that, without the SLH module, the corresponding cellulosome complex may lack the capacity to anchor onto the host microbe’s cell surface (Schwarz, 2001; Bayer et al., 2004). Presence or absence of the SLH module corresponds to the scaffold backbone genes being categorized into three types: type “A1,” type “A2,” and type “A3” according to whether the SLH module is initially in (“A1”) or at least could be assembled into (“A2”) scaffold backbones; and the SLH module is missing in scaffold genes of type “A3”.

Among non-cellulosome gene sets, highlighted in this study is the potential role of enzymes encoded by genes (type “B” genes, hereafter, also termed as SLH-cCBM genes) harboring both the SLH module and the cellulose-binding CBM (cCBM) module, and these SLH-cCBM enzymes may mediate the microbe–cellulose adhesion. Glucanase GH modules (hereafter, termed as cGH) are identified in both type “C” and type “D” putative cellulolytic genes, and type “C” genes also harbor the cCBM modules alongside the cGH modules when such cCBM modules are absent in type “D” genes. Consideration behind the differentiation of type “C” and type “D” genes is that, without the cCBM module, enzymes or subunits of enzymes encoded by type “D” genes are more likely to be part of free cellulases instead of cellulose-binding cellulases, and it has been reported that those free cellulases would contribute little to the microbes’ cellulose-hydrolyzing activity (Bayer et al., 1983; Lynd et al., 2002).

### Genome-Centric Genotype-Based Categorization of Putative Cellulolytic Microbes

**FIGURE 2:**
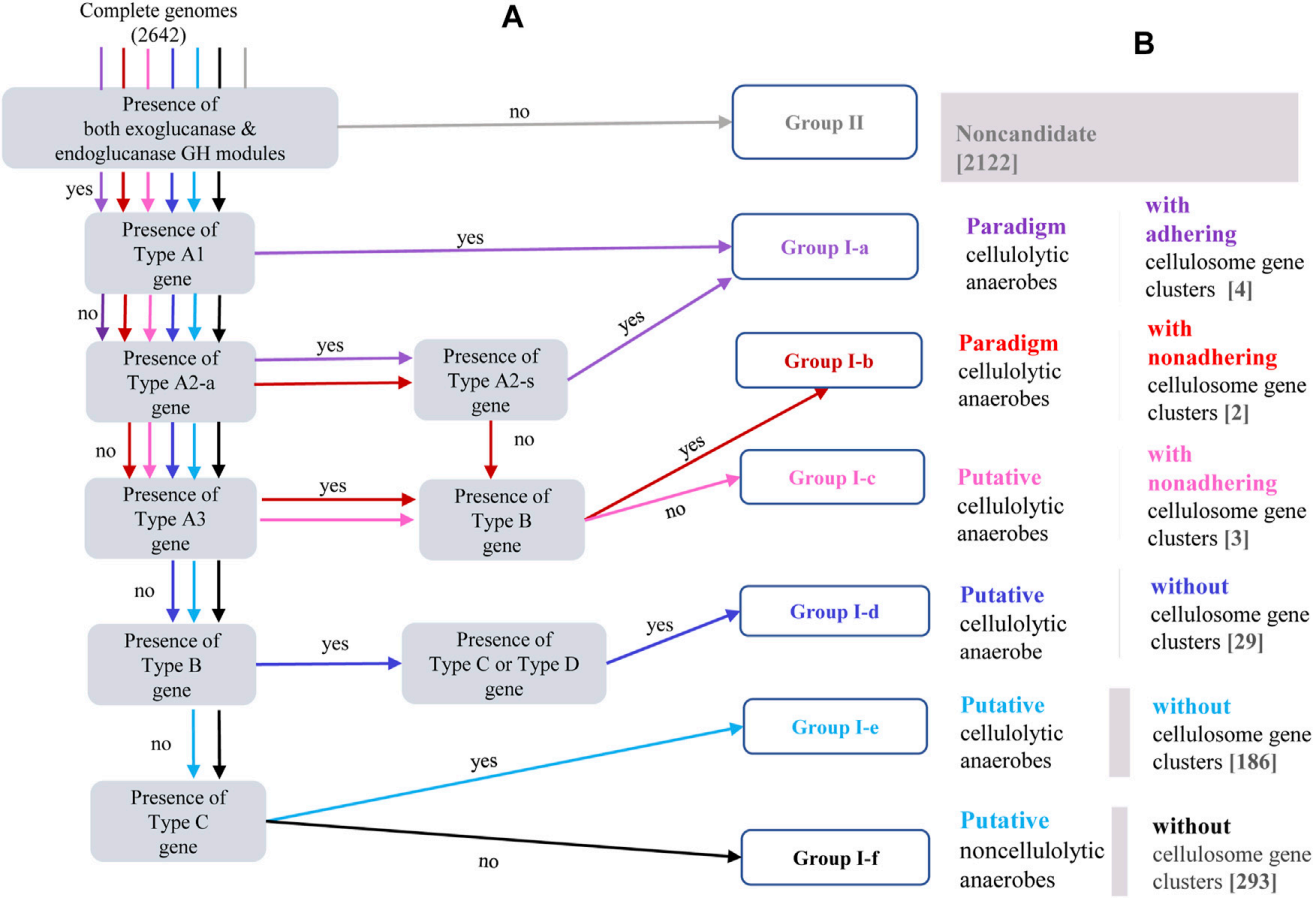
**(A)** Schematic description of the categorization procedure embedded in the bioinformatic pipeline for genome-centric identification and categorization of putative cellulolytic anaerobes. Different colors are allocated to different paths through which genomes of Group I-a [purple], Group I-b [red], Group I-c [pink], Group I-d [blue], Group I-e [sky-blue], Group I-f [black], and Group II [grey] were identified and categorized; in each shaded bracket is one criteria to be checked; genomes meeting one specific criteria will be passed to a downstream criteria led by the “yes” arrow, otherwise, be passed to a downstream criteria led by the “no” arrow; and each genome will be subjected to series of criteria check before this genome would be assigned to a specific group. The gene types **(A1, A2-a, A2-b, A3, B, C and D)** specified in each shaded bracket have been defined in Table 1 and schematically visualized in Figure 2. **(B)** Corresponding phenotype description of each genotype category, numbers in the brackets indicate number of complete genomes assigned into each genotype category.

A genome-centric genotype-based categorization of putative cellulolytic microbes is proposed in this study, and synergy among the carbohydrate-active units were considered in this correlation analysis, that is, synergy among CAZy modules in same genes and synergy among carbohydrate-active genes in the same population. A schematic description of the categorization flow and a summary of the criteria applied could be found in Figure 2 and Table 2. The first feature considered in this genome-centric categorization was the presence of both the exoglucanase GH modules and the endoglucanase GH modules, and genomes annotated with both the exoglucanase GH module and endoglucanase GH module were preliminarily categorized into Group I, otherwise, into Group II.

**TABLE 2:** Genotype-based categorization of genomes annotated with both exoglucanase and endoglucanase GH modules. The cellulose-binding CBM module was referred to as “cCBM,” for example, the SLH-cCBM genes are genes harboring both the SLH module and the cellulose-binding CBM module; the exo/endoglucanase GH modules were referred to as “cGH” modules in this table.

Abundance of different genes identified					Category	Number of genomes assigned in each group
Adhering scaffold (A1 or A2)	Free scaffold (A3)	SLH-cCBM (B)	cGH-cCBM (C)	Solitude GH (D)		
>=1	>=0	>=0	>=0	>=0	Group I-a	4
0	>=1	>=1	>=1	>=1	Group I-b	2
0	>=1	0	>=1	>=1	Group I-c	3
0	0	>=1	>=1	>=1	Group I-d	7
0	0	>=1	>=1	>=0		
0	0	>=1	>=0	>=1		
0	0	0	>=1	>=1	Group I-e	139
0	0	0	>=1	0		
0	0	0	0	>=1	Group I-f	106

**FIGURE 3:**
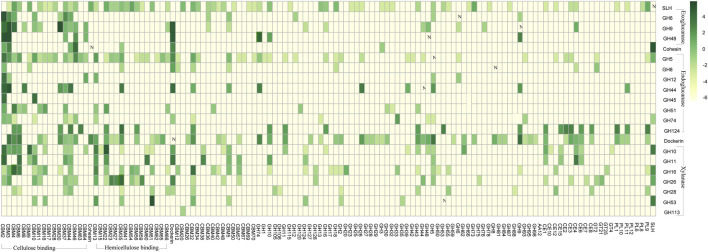
Frequencies of the CAZy modules co-occurring with each other in same genes. Vertically listed are the twenty-one selected CAZy modules, including the four exoglucanase GH modules, the seven endoglucanase GH modules, the seven xylanase GH modules, the cohesion module, the dockerin module, and the SLH module. The CAZy modules lining horizontally are those modules being observed in same genes as at least one of the 21 vertically listed CAZy modules. Co-occurring frequencies are presented in log format-lg (x+0.01) as indicated in the scale bar, and the plain numbers “x” denoting the co-occurring frequencies could also be found in Supplementary Appendix S1.

Presence of the cellulosome gene clusters is considered with the highest priority in further characterization of genomes in Group I. According to whether the SLH module is present in the cellulosome gene clusters, and if not, whether some cellulosome-independent SLH-cCBM genes are present in its genome, genomes annotated with the cellulosome gene clusters were categorized into three genotype groups: Group I-a, Group I-b, and Group I-c. Scaffold genes in Group I-a are of either type “A1” or type “A2,” and these two types of SLH-harboring scaffold genes correspond to cellulosome complexes that are adhesive to host microbe’s cell surface. The scaffold genes in genomes of both Group I-b and Group I-c are of type “A3”, without the cell surface–anchoring SLH module; and SLH-cCBM genes (type B gene) are identified in genomes of Group I-b, while such SLH-cCBM genes are absent in genomes of Group I-c. The proposed role of enzymes encoded by the SLH-cCBM genes has been introduced in previous sections, and for anaerobes in Group I-b, the CEM complex mediated by the SLH-cCBM enzymes may sandwich the cell-free cellulosome complexes in close proximity to both the host microbe cell and its cellulose substrate; there is a high chance that the cellulosome complexes in anaerobes of Group I-c may be in a real “free” state.

Presence of the SLH-cCBM gene (type B gene) is the defining feature in recognizing genomes of Group I-d. Presence of the cGH-cCBM genes (type C gene) is the feature that distinguishes genomes of Group I-e from genomes of Group I-f; and genomes categorized in Group I-f are those annotated with the genotype feature characterized by the property that all of their putative cellulolytic genes are free of the cCBM module (type D gene). Considerations behind categorization of these three genotype groups are 1) in microbes of Group I-d, synergy among carbohydrate-active enzymes in CEM complexes may still be possible with the mediation of the SLH-cCBM enzymes; 2) in microbes of Group I-e, synergy between the cCBM module and the cGH module in the same gene is possible; and 3) while in microbes of Group I-f, although cellulose-active CAZy modules were identified in their genomes, neither of the two types of aforementioned synergy is possible.

## Results

### Co-Occurring Patterns Among Carbohydrate-Active Enzyme Modules in Carbohydrate-Active Genes

Among all the carbohydrate active genes annotated in the 2,642 complete genomes, we specifically focus on genes harboring at least one of the 24 selected CAZy modules that are highly relevant in cellulose hydrolysis (Supplementary Tables S2, S3). These 24 selected CAZy modules include four exoglucanase GH modules, seven endoglucanase GH modules, seven xylanase GH modules, three lytic polysaccharide monooxygenase (LPMO) modules, the cohesion module, the dockerin module, and the SLH module. Considerations behind selection of these 24 CAZy modules are 1) the endoglucanase GH modules are vital components of endoglucanase that randomly cuts the cellulose polysaccharide chain at internal amorphous sites, generating oligosaccharides of various lengths, and the exoglucanase GH modules are vital components of exoglucanase that acts in a processive manner on the ends of the cellulose polysaccharide chains, liberating either glucose or cellobiose as major products (Lynd et al., 2002); 2) the xylanase GH modules mediate the hydrolysis of hemicellulose (Cantarel et al., 2009); 3) the LPMO modules would cleave the 1,4-glycosidic bonds in various polysaccharides and chitin (Busk & Lange, 2015); and 4) the SLH, dockerin, and cohesion modules are essential components of the cellulosome complexes (Bayer et al., 2004).

Such co-occurring frequencies has been summarized in Figure 2. One of the most distinct co-occurring patterns observed was between the endo/exoglucanase GH modules (i.e., cGH, e.g., GH48) and the cellulose-binding CBM modules (i.e., cCBM, e.g., CBM30). As summarized in Supplementary Appendix S2, among the 2,642 complete genomes in which CAZy modules were identified, a total number of 672 genes were annotated with the presence of both the cGH module and the CBM module, and in 635 out of these 672 genes (∼94.5%), at least one of the CBM modules co-occurring with cGH modules is a cellulose-binding cCBM module. Take GH48 for example. Frequencies of GH48 being observed in same genes as CBM2 is 51%, and frequencies of GH48 being observed in same genes as CBM3 is 48.8%. Such high frequencies of cGH modules co-occurring with cCBM module are in accord with the reported importance of cCBM modules in 1) initiation of the exo/endoglucanase’s hydrolytic activity and 2) progressiveness of the exoglucanase along the cellulose chains (Lynd et al., 2002).

One intriguing pattern revealed in this co-occurrence analysis is that, among all the carbohydrate-active genes annotated in these 2,642 microbe strains, there is 0% chance that an SLH module would be identified in the same gene as the dockerin module, which means that, unlike the GH module and the CBM module, the SLH module could not be incorporated into the cellulosome gene clusters through a dockerin partner. The reason why this observation was specially pointed out here is that presence of the SLH module in or the SLH module being able to be incorporated into the cellulosome gene clusters is critical in predicting whether the corresponding cellulosome may adhere onto the host microbes’ cell surface or not. Considering that there is 0% chance that a SLH module would be identified in the same gene as the dockerin module, the SLH module could only be assembled into the scaffold backbone with a cohesion partner, and this is the reason why the “A2-b” gene was defined as a gene that harbors at least one SLH module and at least one cohesion module (rather than a dockerin module), and the corresponding “A2-a” scaffold backbone gene needs to have at least one dockerin module alongside >= 3 cohesion modules. Integration of the dockerin module in “A2-a” with the cohesion module in “A2-b” would assemble the SLH module into type “A2” scaffold backbones.

### Genotype–Phenotype Correlation on the Cellulose-Hydrolyzing Capacity of Anaerobe Strains Categorized Into Different Genotype Groups

How do the microbes’ actual cellulose-hydrolyzing capacity correlate to their genotype features as characterized in this study? By examining the cellulose-hydrolyzing activity reported for each of the microbe strain categorized in each of the genotype group, a genotype–phenotype correlation pattern was derived. A detailed summary of the cellulose-hydrolyzing capacity, aerobic/facultative anaerobic/anaerobic lifestyle, and the taxonomy affiliation of microbe strains categorized into each genotype group can be found in Supplementary Appendix S2.

**FIGURE 4:**
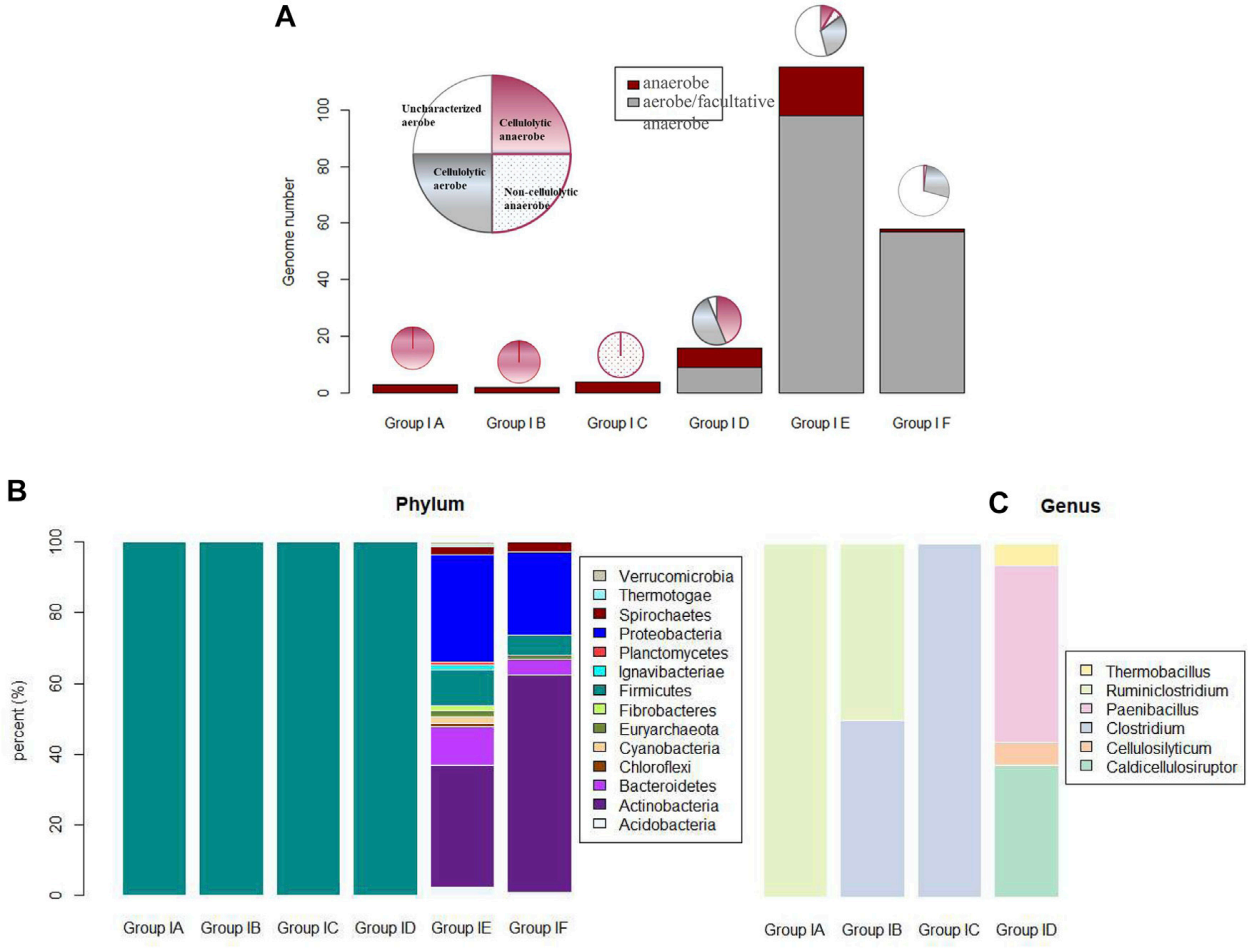
**(A)** Phenotype features (i.e., anaerobic, aerobic, cellulolytic/noncellulolytic/uncharacterized) of microbe strains categorized in six genotype groups (I-a to I-f); **(B)** phylum affiliation of microbe strains in the six genotype groups; and **(C)** genus affiliation of microbe strains in the first 4 genotype groups of I-a to I-d. Due to challenges associated with color assignment for the 114 genera of microbe strains in group I-e and group I-f, genus affiliation of microbe strains in these two genotype groups are not visualized in this figure.

**FIGURE 5:**
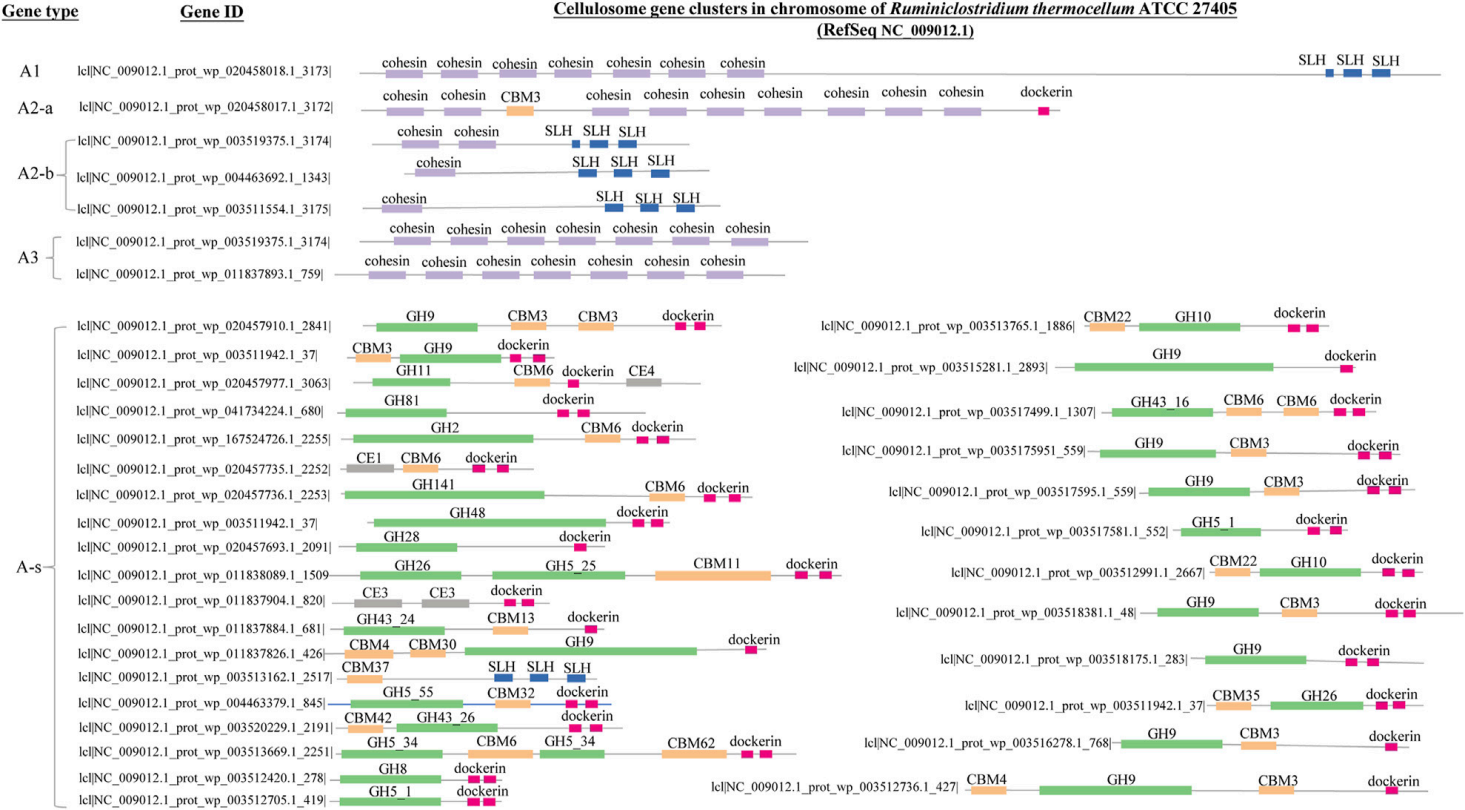
Genes of cellulosome gene clusters in the chromosome (NC_009012.1) of *Ruminiclostridium thermocellum* ATCC 27405. The GH modules, the CBM modules, the SLH module, the dockerin module and the cohesion module are represented with bars in green, yellow, blue, pink and purple, respectively; the width of each bar and its position denote length (bp) of each module and its position along genes. Definition of the different gene types **(A1, A2-a, A2-b, A3, A-s)** are the same as that summarized in Table 1 and Figure 2. The corresponding IDs of all the cellulosome genes have also been provided.

Cellulosome complex and CEM complex formations may elicit synergy among extracellular CAZy enzymes and minimize diffusion loss of the hydrolyzing products; this economic way of enzyme utilization should be crucial for anaerobes to circumvent a luxury production of enzymes in high concentrations as in aerobes (Schwarz, 2001). In accordance with this is the observation as summarized in Figure 4: microbes with cellulosome gene clusters (categorized in Group I-a, I-b, and I-c) are all of anaerobes, and anaerobe strains in these three groups are either of genus *Ruminiclostridium* or *Clostridium*. Take *Ruminiclostridium thermocellum* ATCC 27405 for example. All the genes in its cellulosome gene clusters are summarized in Figure 5; such visualization of the CAZy modules along genes is achieved with the genoplot R script as introduced in the previous section, and a more detailed information on the CAZy module arrangement along all its carbohydrate active genes (includes but are not limited to cellulosome gene clusters) can be found in the Supplementary Appendix S3. In comparison, taxonomy affiliation of microbe strains assigned in Group I-d, I-e, and I-f are much more diverse, covering 114 genera, and aerobes/facultative anaerobes are observed only in these three genotype groups.

We focused mainly on anaerobes for the genotype–phenotype correlation analysis on the corresponding microbes’ cellulose-hydrolyzing capacity. First, in anaerobic environments such as rumen or anaerobic digesters, only anaerobes are the active participants, and only anaerobes are involved in the bioprocessing of cellulosic biomass through fermentation (e.g., butanol fermentation); second, many well-known aerobic cellulose hydrolysers (e.g., *Cytophaga*) have no well-known mechanisms of cellulose hydrolysis (Ji et al., 2012), and as summarized in Figure 4, a high portion (i.e., 72% aerobes in Group I-e and 63% aerobes in Group I-f) of aerobes have not been well-characterized on whether they are cellulolytic or not. So, it is not practical to establish the genotype–phenotype correlation for aerobes based on their cellulose hydrolysis competency.

None of the anaerobe strains categorized in Group II are cellulolytic, which may indicate that the presence of both endoglucanase GH module and exoglucanase GH module is essential for an anaerobe to be cellulolytic. For anaerobe strains that are categorized in Group I, cellulosome gene clusters were identified in nine anaerobe strains of three genotype groups: Group I-a, Group I-b, and Group I-c. The four anaerobe strains of Group I-a, *R. thermocellum* ATCC 27405*, R. thermocellum* DSM 1313, *C. clariflavum* DSM 19732, and *C. cellulovorans* 743B, are all paradigm cellulolytic microorganisms with tethered cellulosome complexes (Feinberg et al., 2011; Ellis et al., 2012; Izquierdo et al., 2012; Nakajima et al., 2017). Both *C. sp.* BNL1100 and *C. cellulolyticum* H10 assigned to Group I-b are reported as proficient cellulose hydrolysers with cellulosome complexes (Giallo et al., 1985; Li et al., 2012). There are three anaerobe strains assigned to Group I-c: *C. acetobutylicum* ATCC 824, *C. acetobutylicum* EA 2018, and *C. acetobutylicum DSM* 1731; all the three *C. acetobutylicum* strains were inert in crystalline cellulose hydrolysis, although cellulosome gene clusters were identified in their genomes (Jacqueline and Belaich, 1985; Mermelstein et al., 1992; Nolling et al., 2001; Yoo et al., 2020)

Anaerobe strains assigned in Group I-d are all of genus *Caldicellulosiruptor* and cellulolytic. There are 18 anaerobe strains assigned in Group I-e. In them, 11 out of 18 anaerobe strains are cellulolytic; potential unique or novel mechanisms for these 11 cellulolytic anaerobe strains to adhere onto their cellulose substrates have to be defined. A typical example of cellulolytic anaerobe in Group I-e is *Ruminococcus albus* 7, a key cellulolytic member of the rumen ecosystem (Melissa et al., 2014). No cellulosome gene cluster was identified in *Ruminococcus albus* 7, although a high number of a total 57 dockerin modules were annotated in its genome.

**FIGURE 6:**
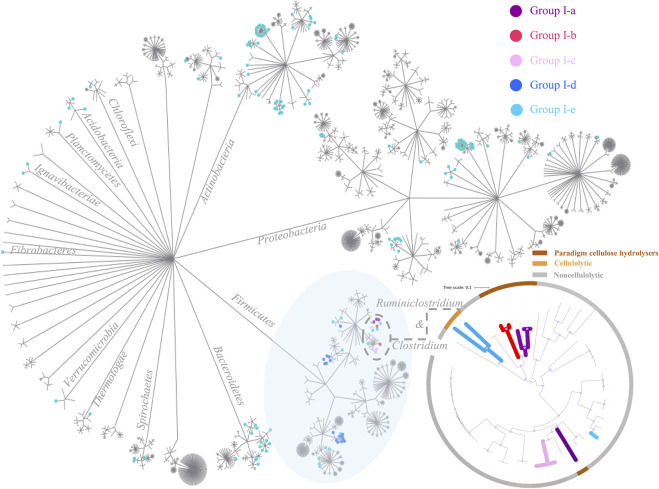
Circle tree of the 2,786 complete genomes. Cellulolytic anaerobes are more likely to be expected in the first five genotype groups (i.e., Group I-a to Group I-e), and nodes of the corresponding strains in these five genotype groups are highlighted in purple, red, pink, blue, and green, respectively. At the lower right corner is a zoomed-in look on microbe strains of *Clostridium* and *Ruminiclostridium*, and this small maximum likelihood phylogenetic tree was constructed using FastTree (Price et al., 2010), based on a set of 120 concatenated universal single-copy proteins (Parks et al., 2018), and visualized trough iTOL (Letunic and Bork, 2021); bootstrap values >0.9 were indicated with blue dots along branches. The inner branches of this smaller tree are highlighted according to the genotype group each strain was assigned to, and the outer ranges were colored according to whether the corresponding strain is cellulolytic or not, that is, brown-paradigm cellulolytic, yellow-cellulolytic, and grey-noncellulolytic anaerobes.

Phylogeny of the 2,786 complete genomes was visualized in a circle tree in Figure 6. One key message delivered in this figure is that the cellulose-hydrolyzing capacity is not phylogenetically conservative, and it is not a reliable approach to predict whether the corresponding microbe is cellulolytic or not based solely on its phylogenetic affiliation, even at the genus level. In comparison, the genotype-based categorization as proposed in this study correlates much better to the corresponding anaerobes’ cellulose-hydrolyzing capacity reported. Take the 40 strains in the genus of *Clostridium* and *Ruminiclostridium* for example. There are four paradigm cellulolytic strains assigned in Group I-a, two paradigm cellulolytic strains assigned in Group I-b, two out of the three strains in Group I-e are cellulolytic, and all the other 32 strains in these two genera are noncellulolytic.

### Scalable Annotation of Large Genome Data by Applying the Bioinformatic Pipeline Developed in This Study

Apart from the annotation of the 2,786 complete genomes as introduced in previous sections, applicability of this pipeline in scalable annotation of the large genome dataset was further evaluated with the annotation of 7,904 reference genomes (this dataset includes metagenome-assembled genomes, MAGs) downloaded from NCBI. Starting with the dbCAN annotation result, applying the pipeline developed in this study, it took ∼30 min to 1) generate a summary file on the diversity and abundances of all the CAZy modules identified in each of these 7,902 genomes, 2) characterize the carbohydrate-active genes according to the clustering patterns of CAZy modules they harbor and count, and 3) identify and categorize genomes of putative cellulolytic anaerobes into the six genotype groups (Supplementary Appendix S4). Among the 7,902 reference genomes annotated, five were assigned into Group I-a, nine in Group I-b, 15 in Group I-c, 3 in Group I-d, 15 in Groups I-e, and eight in Group I-f. Supplementary Figure S1 presents the phylogeny of these reference genomes assigned into the first five genotype groups. Consistent with results of the survey on the 2,786 complete genomes, cellulosome gene clusters were annotated only in a small number (29 out of 7,902) of reference genomes, and the genotype categories were not phylogenetically conservative. Although the majority of these reference genomes are of no pure culture isolates, which means that no phenotype documentation is available for the corresponding microbes’ cellulose-hydrolyzing competency, still, we observed that, among those reference genomes categorized to be of putative cellulolytic anaerobes, especially those of group I-a, I-b, and I-c, they are genomes/draft genomes reconstructed from anaerobic consortia for cellulosic biomass degradation or genomes reconstructed from the rumen or gut microbiota. A cautiously positive estimation here is that all those reference genomes categorized in Group I-a and I-b should be of paradigm cellulolytic anaerobes, although no pure culture of the corresponding anaerobe strain is available yet.

## Discussion

### Why *C. acetobutylicum* Strains Are Inert in Cellulose Hydrolysis, Although Cellulosome Gene Clusters Are Annotated in Their Genomes?

It has been a long-standing enigma why the *C. acetobutylicum* strains are inert in cellulose hydrolysis, although cellulosome gene clusters are identified in their genomes (Fabrice et al., 2002). *C. acetobutylicum* strains are well-known in the industrial butanol–acetone fermentation from starch, the Fernbach–Weismann patent (Sreekumar et al., 2015); it would be of high environmental and ecnomic interest if *C. acetobutylicum* strains could also ferment the much cheaper cellulosic biomass to produce butanol and acetone.

A zoomed-in look on the cellulosome gene clusters in *C. acetobutylicum* strains revealed a potential explanation on their inertness toward cellulose: the scaffold genes in the cellulosome gene clusters of *C. acetobutylicum* strains are of type “A3”, which indicates that they are in lack of the SLH module, and the corresponding cellulosome complexes may be in a “free” state instead of being cell surface adhering. Unless alternative machinaries exist that could facilitate the cellulose–microbe adhesion, CEM complex formation may not be possible for these *C. acetobutylicum* strains. Similar to extracellular free cellulases (Bayer et al., 1983; Raphael and Bayer, 1983), free cellusomal complexes that could not be held in proximity to both the microbe cell and the cellulosic substrate might limit the host microbes’ access to hydrolyzing products, in which case, the host microbe might become reluctant in energy-consuming synthesis and assembly of the cellulosome complexes. This may possibly be the underlying reason why C. *acetobutylicum* strains are inert in cellulose hydrolysis although cellulosome gene clusters are identified in their genomes.

Although the presence of the cellulosome gene clusters has always been taken as an indicator of the corresponding model cellulolytic microbes, a closer interrogation in this study signified the necessity of a finer categorization of the cellulosome complexes, according to whether they are of the capacity to adhere onto the host microbes’ cell surface or not. Cellulosome complexes missing the SLH module may not be able to adhere onto the host microbes’ cell surface to initiate the formation of the CEM complexes. In other words, although cellulosome complexes by its nature could enable the assembly of a number of carbohydrate-active units, they do not certainly guarantee the CEM complex formation. Also, this obervation also points to one possible genome engineering strategy to make the C. *acetobutylicum* strains cellulose-active, by either introducing the SLH module into its cellulosome gene cluster or by introducing into its chromosome/plasmid, some gene-encoding enzymes that could mediate cellulose–microbe adhesion, and the latter will be discussed in detail in the following section.

### Potential Role of Surface Layer Homology: Cellulose-Binding CBM Module Enzymes in Mediating the Cellulose–Enzyme–Microbe Complex Formation in Cellulolytic Microbes

In characterizing the cellulose-hydrolyzing activity of *Caldicellulosiruptor bescii* DSM 6725 (categorized to Group I-d in this study), Dam et al. observed that *C. bescii* cells directly attached to its cellulosic substrate (i.e., switchgrass) although this strain did not produce a cellulosome complex (Dam et al., 2011). They suggested in their research article that SLH modules might involve in the cell-substrate interactions by mediating the binding of proteins to the microbial cell surface, and genes harboring both the SLH module and the cCBM module (e.g., genes with SLH module combined with the GH5 and CBM28 modules) are among several candidate genes they predicted that may encode the cell-adhering proteins. This is the first time that the potential role of enzymes encoded by SLH-cCBM genes in mediating microbe–cellulose adhesion was hinted.

In this study, the putative role of enzymes encoded by the SLH-cCBM genes in mediating the microbe–cellulose adhesion is derived from the cellulolytic activity of anaerobes in Group I-b and Group I-d. Specifically, same as that in the *C. acetobutylicum* strains (of Group I-c), the cellulosome gene clusters in *C. cellulolyticum* H10 and *C.* BNL1100 (both of Group I-b) are also free of the SLH module, while the differentiating feature between genomes in group I-b (e.g., *C. cellulolyticum* H10) and genomes in group I-c (e.g., C. *acetobutylicum*) is that several cellulosome-independent SLH-cCBM genes are identified in the former. In contrast to the *C. acetobutylicum* strains (Group I-c) being inert in cellulose hydrolysis, cellulosome complexes formation and high cellulose-hydrolyzing activity were observed in both *C. cellulolyticum* H10 and *C.* BNL1100 (Group I-b) (Giallo J., 1985; Li et al., 2012). Similarly, genomes of group I-d and group I-e are also differentiated by whether SLH-cCBM genes are present in their genomes; owing to the presence of the SLH-cCBM genes, anaerobes of Group I-d were cellulolytic, with direct microbe-cellulose attachment observed (Dam et al., 2011); while cellulolytic activity of anaerobes in group I-e varied.

We proposed in this study that the CEM complex formation mediated by enzymes encoded by SLH-cCBM genes might be essential for the cellulolytic activity of anaerobes without cellulosome gene clusters (i.e., Group I-d) and of anaerobes whose cellulosome gene clusters are in lack of the SLH module (i.e., Group I-b). Future wet-lab experiments are needed for a rigorous verification on the functional roles of these SLH-cCBM enzymes in mediating the proximity between the anaerobe cell and its cellulosic substrates, and a confirmation on this would highlight a potential genome engineering strategy to make some non-cellulolytic strains cellulolytic, for example, the well-known *C. acetobutylicum* strains (Sreekumar et al., 2015).

### Limitations of the Annotation Approach Developed in This Study

One of the key messages delivered in this study is that, in predicting whether the corresponding microbe is of cellulose-hydrolyzing activity or not, identification of the machineries that may mediate the microbe–cellulose adhesion and the CEM complex formation is equally important as the identification of individual carbohydrate-active units. The pipeline developed in this study is based on a CAZy annotation platform, which means that non-CAZy enzymes that may mediate the microbe-cellulose adhesion could not be covered by this annotation approach. For example, it was reported that the glycocalyx-containing extracellular polymeric substances might have acted as a “glue” between the microbe cell and its cellulosic substrates in *R. albus* 7 (Weimer et al., 2006); it was also reported that the fibronectin type 3-like (Fn3) domains contain binding sites for the cell surface (http://pfam.sanger.ac.uk), and those lysine motif (LysM) domains in a variety of enzymes that are involved in bacterial cell wall degradation may also have a general peptidoglycan-binding function (Bernard et al., 1992). However, current knowledge on such alternative “glue” is a bit too limited to be leveraged for automatic gene-/genome-centric recognition of them. This limitation leads to uncertainty in predicting the cellulose-hydrolyzing capability of microbes assigned into Group I-e and Group I-c, and for anaerobe strains that are of confirmed cellulose hydrolyzing activities in Group I-e or Group I-c, for example, *R. champanellensis* 18P13 (Chassard et al., 2012), potential novel microbe-cellulose adhesion mechanisms await to be uncovered. It should also be noted that, same as that in other genome-centric analysis, the quality of the genomes matters, and more reliable function interpretation is expected for genomes with higher completeness and lower contamination.

Another limitation associated with this CAZy-based annotation comes from the promiscuity of the CAZy families. Some CAZy families are of multiple biochemical functions; for example, although the GH9 module predominantly corresponds to endoglucanase activity (EC 3.2.1.4), in a few cases it also corresponds to exoglucanase activity (EC 3.2.1.91); similar promiscuity is also noted for the GH5 module. In this study, we did not apply a subdivision of each CAZy module through further annotation at the EC (enzyme commission) level; and as long as exoglucanase activity has been documented for a specific GH module, we would count this GH module as a GH module with exoglucanase activity. Considerations behind this tactic are 1) comparing with the enzyme commission numbers (EC), CAZy is more often employed in the analysis of the microbial metabolism against carbohydrates, and 2) although a finer subdivision of each CAZy module may always contribute to a higher resolution, a lack of confidence in current genome-centric prediction of the corresponding anaerobes’ cellulolytic competency is mainly due to a failure in recognizing the synergy mechanisms among annotated CAZy units.

In summary, we fully acknowledge that there is room for further improvement of this CAZy-based annotation approach for genome-centric interpretation on the specific functional niche of cellulose hydrolysis for anaerobes; nevertheless, it is also fair to say that the two limitations as discussed above are not originated from the methodologies applied in this study; rather, they are largely associated with the corresponding knowledge gaps awaiting to be addressed in the field. In this study, by introducing a set of standards to recognize the synergy among varying carbohydrate-active units, an explicit genotype–phenotype correlation was evidenced to be feasible for cellulolytic anaerobes. We believe the genotype–phenotype correlation pattern is revealed in this study, and the pipeline developed in this study would be a good add as a bioinformatic tool for genome-centric interpretation of uncultivated anaerobes, specifically on their functional niche of cellulose hydrolysis.

## Code Availability

All scripts written for this study are available at https://github.com/yuboer/genome-centric-portrait-of-cellulose-hydrolysis. A test dataset has also been provided in Github to showcase the usage of the pipeline developed in this study. R scripts for the MAG analysis has also been made into a docker container image, which can be found in the “for_docker_image” folder.

## Data Availability

The original contributions presented in the study are included in the article/Supplementary Material; further inquiries can be directed to the corresponding author.

## Abbreviations

CAZy, carbohydrate active enzyme; CBM, carbohydrate binding module; cCBM, cellulose-binding CBM module; CUPP, conserved unique peptide patterns; CEM complex, cellulose-enzyme-microbe complex; cGH, cellulose-active glycoside hydrolase; EPS, extracellular polymeric substances; GH, glycoside hydrolase; LPMO, lytic polysaccharide monooxygenases; MAGs, metagenome assembled genomes; NGS, next generation sequencing; SLH, surface layer homology.
