# Supplementary material for: Reliable and Scalable Identification and Prioritization of Putative Cellulolytic Anaerobes With Large Genome Data
Source: Front Bioinform. 2022 Mar 17;2:813771. doi: 10.3389/fbinf.2022.813771 (PMC9580877; doi:10.3389/fbinf.2022.813771)
Supplement: Supplementary file 2 [file DataSheet5.docx]

**Supporting Information**

**Title:** **Reliable and scalable identification and prioritization of putative cellulolytic anaerobes with large genome data**

**Authors:** Yubo Wang^1^, Liguan Li^1^, Yu Xia^1,2^, Tong Zhang^1,3,4*^

**Author affiliation:**

1. Environmental Microbiome Engineering and Biotechnology Laboratory, The University of Hong Kong, Hong Kong SAR, China.

2. School of Environmental Science and Engineering, Southern University of Science and Technology, Shenzhen, China

3. School of Environment and Energy, Peking University Shenzhen Graduate School, Shenzhen, China.

4. Shenzhen Bay Laboratory, Shenzhen, China.

*** Corresponding author:**

Address: Environmental Microbiome Engineering and Biotechnology Laboratory, The University of Hong Kong, Pokfulam Road, Hong Kong

Tel: 852-28591968 (lab)4, 28578551 (office)

Fax: 852-25595337

E-mail: [zhangt@hku.hk](mailto:zhangt@hkucc.hku.hk)

Table S1. Preliminary categorization of the 2642 complete genomes

| **Grouping**  **No.** | **Number of GH modules with different activities** | | | | **Number of genomes** |
| --- | --- | --- | --- | --- | --- |
|  | Exoglucosidase | Endoglucosidase | Hemicellulase | Oligosaccharidase |  |
| Group I | >=1 | >=1 | >=0 | >=0 | 510 |
| Group II | >=1 | 0 | >=0 | >=0 | 101 |
|  | 0 | >=1 | >=0 | >=0 | 605 |
|  | 0 | 0 | >=1 | >=0 | 432 |
|  | 0 | 0 | 0 | >=1 | 532 |
|  | 0 | 0 | 0 | 0 | 352 |

As has been summarized in Table S1, among the 2642 complete genomes investigated, 210 of them harbor both exoglucanase and endoglucanase GH modules, being preliminarily categorized into Group I. It was noted that a total number of <=2 cGH modules would be identified in a genome if this genome was annotated with only the exoglucanase GH module or with only the endoglucanase GH module, and none of these genomes are of anaerobe strains with reported cellulolytic activities. Cellulolytic anaerobes are only to be expected in Group I. for example, among the 2122 complete genomes assigned in Group II, adhering scaffold genes were identified in *Desulfotomaculum acetoxidans* DSM 771, without the presence of both the exoglucanase GH modules and the endoglucanase GH modules, *Desulfotomaculum acetoxidans* DSM 771 is incapable of utilizing cellulose.

Table S2. Summary of the GH modules with reported exoglucanase activity, endoglucanase activity, lytic polysaccharide monooxygenases activity, xylanase activity and oligosaccharidase activity, respectively

| **Cellulase** | | **Hemicellulase** | | **Oligosaccharidase** | |
| --- | --- | --- | --- | --- | --- |
| GH module | Known activity | GH  module | Known activity | GH  module | Known activity |
| GH6 | endo/exoglucanase | GH10 | endo-1,4-ß-xylanase | GH1 | ß-glucosidase, exo-ß-1,4-glucanase,  ß-xylosidase, ß-galactosidase, ß-mannosidase |
| GH9 | endo/exoglucanase | GH11 | endo-1,4-ß-xylanase | GH2 | ß-galactosidase |
| GH48 | endo/exoglucanase | GH16 | xyloglucanase | GH3 | ß-glucosidase, exo-ß-1,3-1,4-glucanase,  1,4-ß-xylosidase |
| GH5 | Endo/exoglucanase | GH26 | ß-mannanase; xylanase | GH29 | ɑ-fucosidase |
| GH7 | endoglucanase | GH28 | galacturonase | GH35 | ß-galactosidase |
| GH8 | endoglucanase | GH53 | endo-1,4-ß-galactanase | GH38 | ɑ-mannosidase |
| GH12 | endoglucanase | G113 | ß-mannanase | GH39 | ß-xylosidase |
| GH44 | endoglucanase | GH134 | ß-mannanase | GH42 | ß-galactosidase |
| GH45 | endoglucanase | GH30 | endo-1,4-ß-xylanase; ß-glucosidase;  ß-xylosidase | GH47 | ɑ-mannosidase |
| GH51 | endoglucanase | GH43 | ß-xylosidase, endo-1,4-ß-xylanase,  arabinanase | GH52 | ß-xylosidase |
| GH61 | endoglucanase | GH98 | endo-1,4-ß-xylanase | GH59 | ß-galactosidase; galactocerebrosidase |
| GH74 | endoglucanase |  |  | GH92 | ɑ-mannosidase |
| GH124 | endoglucanase |  |  | GH94 | cellubiose phosphorylase |
| AA11 | lytic polysaccharide monooxygenases |  |  | GH116 | ß-galactosidase; ß-xylosidase |
| AA10 | lytic polysaccharide monooxygenases |  |  | GH120 | ß-xylosidase |
| AA9 | lytic polysaccharide monooxygenases |  |  | GH125 | exo-a-1,6-mannosidase |

Table S3. Summary of the CBM modules with cellulose-binding activity, hemicellulose-binding activity, glycogen-binging activity and chitin-binding activity, respectively.

| **Cellulose-binding** | **Hemicellulose-binding** | **Glycogen-binding** | **Chitin-binding** |
| --- | --- | --- | --- |
| CBM2 | CBM9 | CBM20 | CBM5 |
| CBM3 | CBM13 | CBM21 | CBM18 |
| CBM6 | CBM15 | CBM25 | CBM19 |
| CBM7 | CBM22 | CBM26 | CBM50 |
| CBM8 | CBM23 | CBM34 | CBM55 |
| CBM9 | CBM24 | CBM48 |  |
| CBM10 | CBM27 | CBM53 |  |
| CBM11 | CBM29 | CBM69 |  |
| CBM16 | CBM31 | CBM67 |  |
| CBM17 | CBM35 | CBM32 |  |
| CBM28 | CBM39 | CBM42 |  |
| CBM30 | CBM41 |  |  |
| CBM37 | CBM43 |  |  |
| CBM44 | CBM44 |  |  |
| CBM46 | CBM52 |  |  |
| CBM49 | CBM54 |  |  |
| CBM63 | CBM56 |  |  |
| CBM64 | CBM59 |  |  |
| CBM4 | CBM60 |  |  |
| CBM1 | CBM61 |  |  |
|  | CBM62 |  |  |
|  | CBM65 |  |  |
|  | CBM66 |  |  |


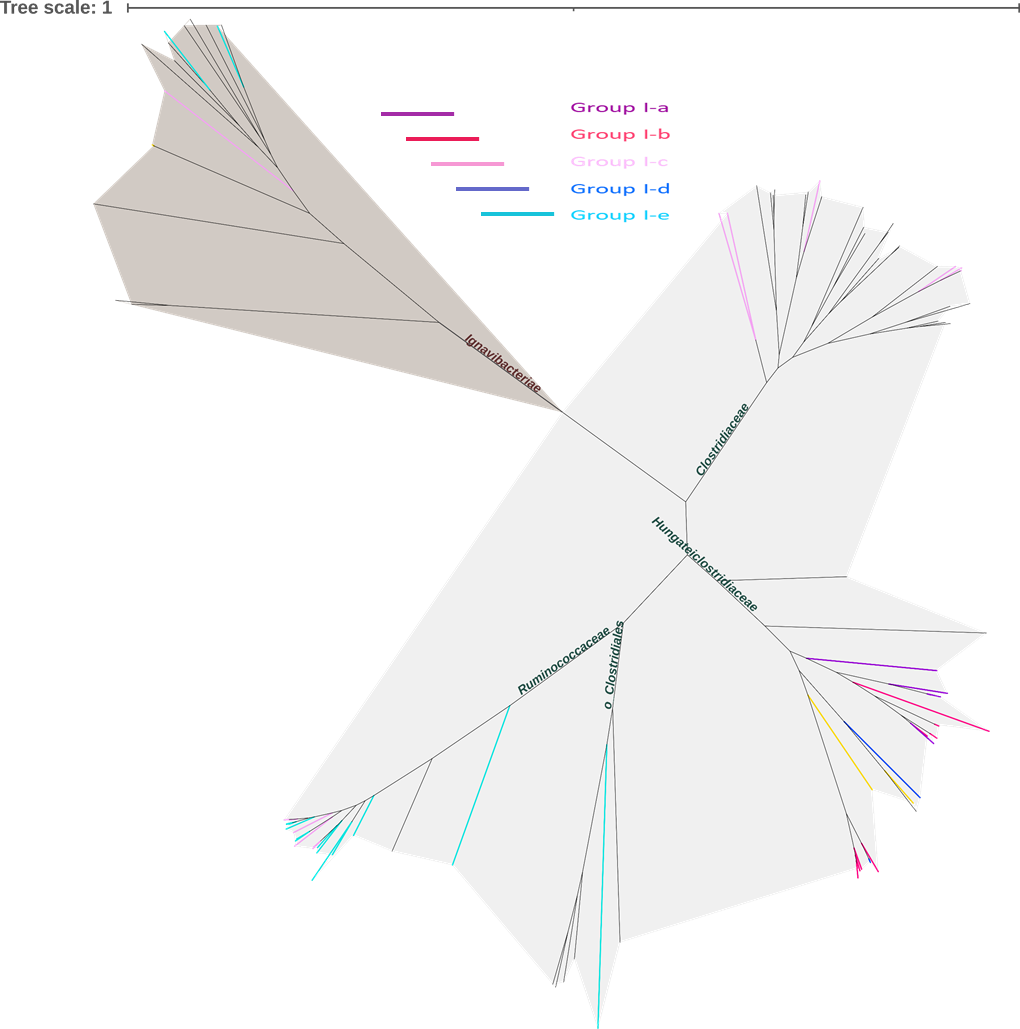


Figure S1. Maximum likelihood tree of putative cellulolytic anaerobes annotated among the 7904 reference genomes. Branches and nodes of genomes categorized into the 5 genotype groups were highlighted in purple, red, pink, blue and green, respectively. The grey branches and grey nodes are of those reference genomes that are phylogenetically close, among the 7904 MAGs, to at least one of these highlighted reference genomes in the 5 genotype groups. The tree was constructed using mafft alignment (1) with 100 bootstraps based on a set of 120 concatenated universal single-copy proteins annotated in each genome (2)

**References**

1. Katoh K, Standley DM. MAFFT multiple sequence alignment software version 7: improvements in performance and usability. Mol Biol Evol. 2013;30(4):772-80.

2. Parks DH, Chuvochina M, Waite DW, Rinke C, Skarshewski A, Chaumeil PA, et al. A standardized bacterial taxonomy based on genome phylogeny substantially revises the tree of life. Nat Biotechnol. 2018;36(10):996-1004.
