# Supplementary material for: Reliable and Scalable Identification and Prioritization of Putative Cellulolytic Anaerobes With Large Genome Data
Source: Front Bioinform. 2022 Mar 17;2:813771. doi: 10.3389/fbinf.2022.813771 (PMC9580877; doi:10.3389/fbinf.2022.813771)

Arrangement of the CAZy modules in NC_009012

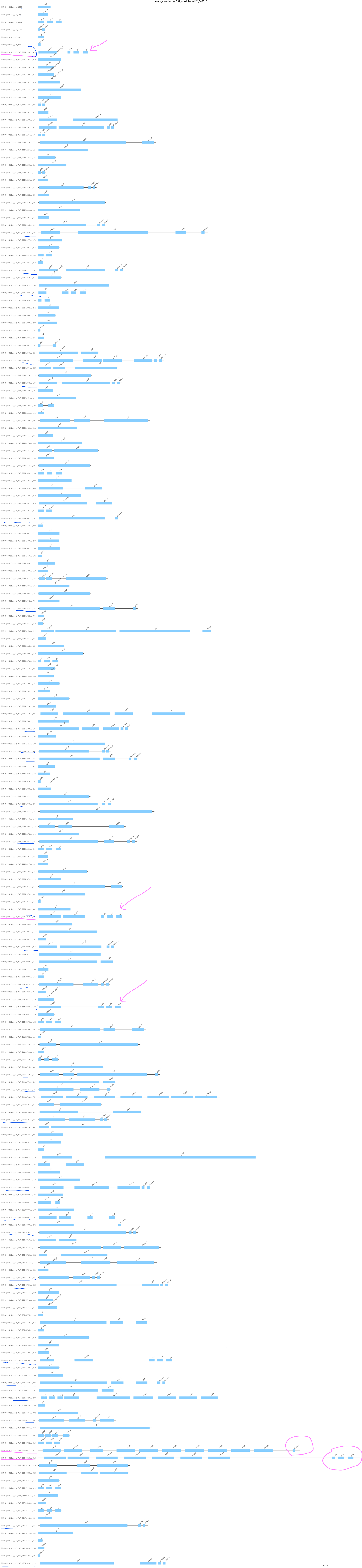

Supplement: Supplementary file 3 [file DataSheet3.PDF]
